# Diffusion-weighted MRI distinguishes Parkinson disease from the parkinsonian variant of multiple system atrophy: A systematic review and meta-analysis

**DOI:** 10.1371/journal.pone.0189897

**Published:** 2017-12-29

**Authors:** Sweta Bajaj, Florian Krismer, Jose-Alberto Palma, Gregor K. Wenning, Horacio Kaufmann, Werner Poewe, Klaus Seppi

**Affiliations:** 1 Department of Neurology, Medical University Innsbruck, Innsbruck, Austria; 2 Dysautonomia Center, Department of Neurology, New York University School of Medicine, New York, New York, United States of America; 3 Neuroimaging Research Core Facility, Medical University Innsbruck, Innsbruck, Austria; Universitat Ulm, GERMANY

## Abstract

**Background:**

Putaminal diffusivity in brain magnetic resonance diffusion-weighted imaging (DWI) is increased in patients with the parkinsonian variant of multiple system atrophy (MSA-P) compared to Parkinson disease (PD) patients.

**Purpose:**

We performed a systematic review and meta-analysis to evaluate the diagnostic accuracy of DWI to distinguish MSA-P from PD.

**Methods:**

Studies on DWI were identified through a systematic PubMed and Clarivate Analytics^®^ Web of Science^®^ Core Collection search. Papers were selected based on stringent inclusion criteria; minimum requirement was the inclusion of MSA-P and PD patients and documented true positive, true negative, false positive and false negative rates or overall sample size and reported sensitivity and specificity. Meta-analysis was performed using the hierarchical summary receiver operating characteristics curve approach.

**Results:**

The database search yielded 1678 results of which 9 studies were deemed relevant. Diagnostic accuracy of putaminal diffusivity measurements were reported in all of these 9 studies, whereas results of other regions of interest were only reported irregularly. Therefore, a meta-analysis could only be performed for putaminal diffusivity measurements: 127 patients with MSA-P, 262 patients with PD and 70 healthy controls were included in the quantitative synthesis. The meta-analysis showed an overall sensitivity of 90% (95% confidence interval (CI): 76.7%-95.8%) and an overall specificity of 93% (95% CI: 80.0%-97.7%) to distinguish MSA-P from PD based on putaminal diffusivity.

**Conclusion:**

Putaminal diffusivity yields high sensitivity and specificity to distinguish clinically diagnosed patients with MSA-P from PD. The confidence intervals indicate substantial variability. Further multicenter studies with harmonized protocols are warranted particularly in early disease stages when clinical diagnosis is less certain.

## Introduction

Parkinson disease (PD) and multiple system atrophy (MSA) are both progressive, neurodegenerative synucleinopathies. Depending on the predominant motor deficits, MSA is sub-divided into a parkinsonian (MSA-P) and a cerebellar (MSA-C) variant. Because MSA-P and PD share several signs and symptoms, they may be mistaken for one another on clinical examination [1] with diagnostic error rates at the first clinical visit reaching 24%. [2] Thus, an early and reliable diagnostic marker is a major unmet medical need. In recent years, several brain magnetic resonance imaging (MRI) features have been described as specific for MSA and as helpful in the differential diagnosis of parkinsonian syndromes. These include atrophy of the putamen, pons, cerebellum and middle cerebellar peduncle (MCP), a dilated fourth ventricle, and various signal intensity alterations on routine MRI in MSA [3–5] whereas conventional MRI is typically normal in PD. Diffusion-weighted imaging (DWI) is of particular interest since it may serve as a quantifiable surrogate marker of neurodegeneration in MSA patients. [6] In fact, increased putaminal diffusivity in DWI is considered a common and diagnostically valuable finding in patients with MSA. [7,8] Here, we present a systematic review and meta-analysis of the diagnostic accuracy of DWI in distinguishing MSA-P from PD.

## Patients and methods

Studies on DWI were identified by two raters (SB, FK) through a systematic PubMed and Clarivate Analytics^®^ Web of Science^®^ Core Collection search. The following search term was used: (“multiple system atrophy” OR MSA OR “olivopontocerebellar atrophy” OR OPCA OR “striatonigral degeneration” OR SND OR “Shy-Drager syndrome”) AND (“magnetic resonance imaging” OR MRI OR diffusion* OR diffusivity* OR DWI OR DTI) (S1 File, Search strategy). The term diffusivity used in this article includes Trace(D), averaged ADCs and mean diffusivity (MD). Full papers published from March 1986 through June 29, 2017 were considered. For further analysis papers had to satisfy the following, predefined eligibility criteria: (1) Papers were required to be published in English or German language. (2) MSA-P and PD patients were included in the study. (3) Studies were required to either report true positive, true negative, false positive and false negative rates or overall sample size and sensitivity and specificity values. Our meta-analysis complied with the Preferred Reporting Items for Systematic Reviews and Meta-Analyses (PRISMA) statement [9] (S1 Checklist, Prisma Checklist).

The risk of bias in individual studies and across studies was performed with a tool for the quality assessment of studies of diagnostic accuracy (QUADAS) [10] (S1 Table, Quadas). The rating was performed by two independent raters (SB, FK) and discordant ratings were resolved in a discussion of the two initial raters and one additional uninvolved senior investigator. The QUADAS questionnaire includes fourteen items covering the following issues: reference standard, covered patient spectrum, verification bias, disease progression bias, review bias, incorporation bias, clinical review bias, test execution, indeterminate results and study withdrawals. Data extraction was done for each paper by the two independent investigators. For statistical analysis the following data were extracted from each of the studies: (1) Number of participants in each group, (2) sensitivity and specificity, or alternatively, true positive, true negative, false positive and false negative rates. Overall sensitivity and specificity were calculated using the hierarchical summary receiver operating characteristics (HSROC) curve approach as described previously [11] and, in addition, both, a summary estimate which includes 95% confidence region and a forecast of the sensitivity and specificity which includes a 95% prediction region, are provided. In this method, the relationship between logit-transformed sensitivity and specificity in each study is quantified by the log diagnostic odds ratio (OR) and the results are used to estimate a summary ROC curve. Between-study heterogeneity was assessed by I^2^ statistic, a parameter that provides a measure of the degree of inconsistency across studies describing the percentage of total variation attributable to heterogeneity, rather than chance. I^2^ values up to 30%–40% are considered as low and up to 50%–60% as moderate heterogeneity. [12] Statistical analysis was performed using STATA (StataCorp 2007, Stata Statistical Software, Release 14.1; StataCorp LP, College Station, TX) exploiting the commands METANDI and MIDAS.

## Results

**Fig 1:**
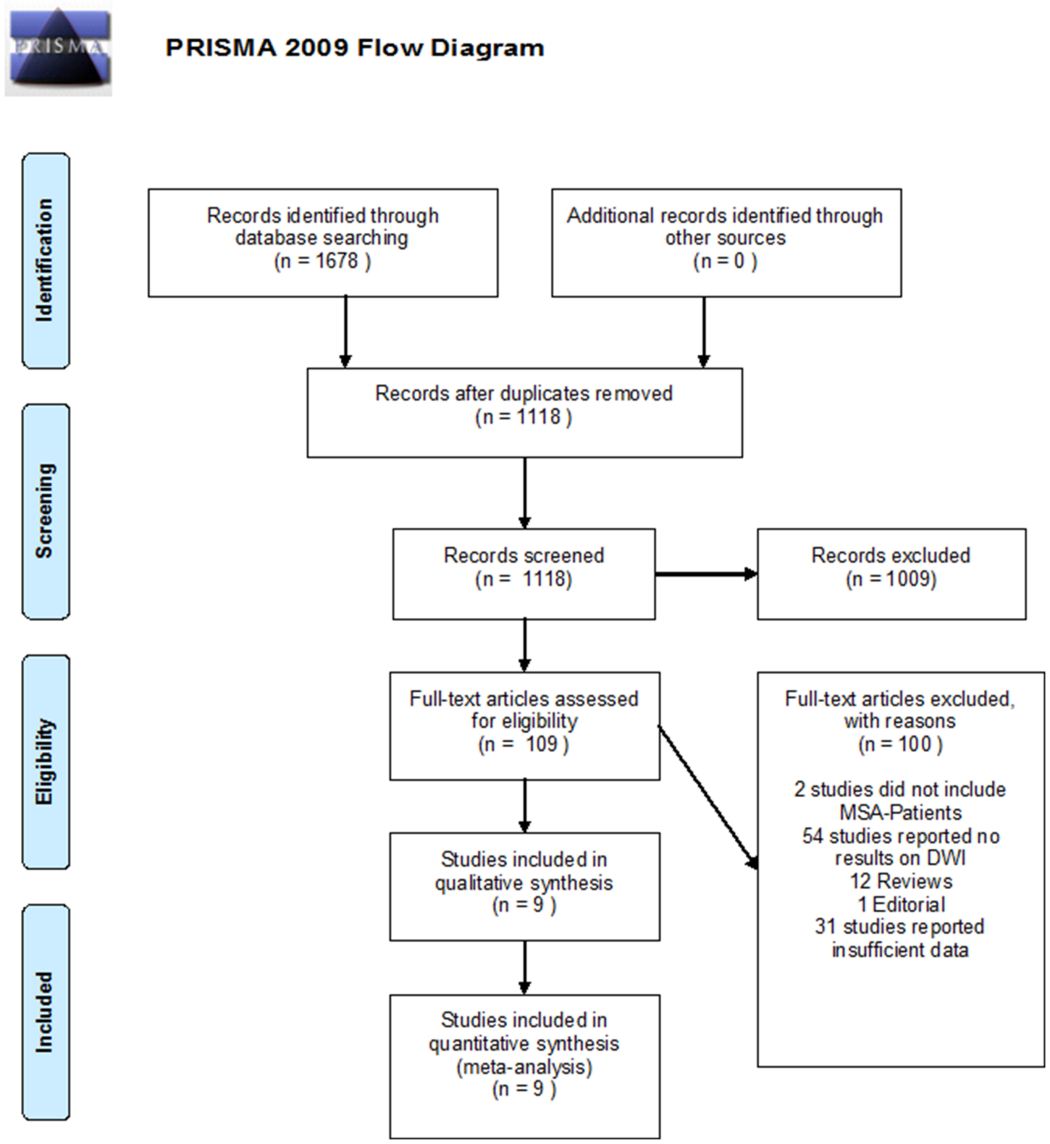
Prisma 2009 flow diagram showing an overview of study selection.

A total of 1678 papers were identified by the initial PubMed and Clarivate Analytics^®^ Web of Science^®^ Core Collection search. After review of the abstracts, and removal of 1118 duplicates, 109 publications were selected for further review of the full texts. Only 9 studies satisfied the predefined criteria and were deemed relevant. A detailed flow chart of the review process is shown in Fig 1. The characteristics of the nine studies [13–21] included in this study are presented in Table 1.

**Table 1:** Overview of eligible studies evaluating diffusivity in different brain regions.

First author	Examined region	n				MSA-P vs. PD		Age (years)			Disease Duration		MRI		
		total	MSA-P	PD	HC	Sensitivity	Specificity	Mean (SD)			Mean (SD)				
Putamen								MSA-P	PD	HC	MSA-P	PD	magnetic field	slice thick ness	Inter-slice gap
**Baudrexel et al. 2013**	posterior putamen*	38	11	13	6	72.7	100.0	66.1 (11.7)	66.8 (8.0)	NA	3.6 (2.2)	6.4 (6.0)	3T	NA	NA
**Baudrexel et al. 2013**	anterior putamen	38	11	13	6	NA									
**Umemura et al. 2013**	Putamen*	138	20	118	NA	85.0	89.0	64.6 (8.2)	60.8 (9.9)	NA	3.6 (1.8)	6.8 (4.9)	1.5T	5 mm	0.5 mm
**Chung et al. 2009**	posterior putamen*	32	10	12	10	66.7	80.0	63.6 (8.25)	65.7 (10.88)	62.1 (9.77)	2.0 (1.1)	2.5 (1.8)	1.5T	6 mm	1 mm
**Köllensperger et al. 2007**	Putamen*	18	9	9	NA	100.0	100.0	66.6 (8.0)	68.1 (4.6)	NA	6.4 (2.4)	11.3 (6.1)	1.5T	3 mm	0 mm
**Ito et al. 2007**	Putamen*	61	20	21	20	70.0	63.6	61.0 (9)	62.0 (11)	62.0 (11)	4.0 (2.0)	10.0 (8.0)	3T	2 mm	0.6 mm
**Nicoletti et al. 2006**	Putamen*	63	16	16	15	100.0	100.0	64.7 (5.1)	61.0 (7.7)	67.5 (6.0)	4.9 (4.0)	7.5 (5.8)	1.5T	5 mm	1 mm
**Sako et al. 2016**	Putamen*	47	11	36	NA	82.0	81.0	60.0 (7.3)	61.0 (8.2)	NA	3.1 (2.3)	4.7 (4.5)	1.5T and 3T	6 mm	1.5 mm
**Seppi et al. 2004**	Striatum*	32	15	17	8	93.0	100.0	63.9 (5.6)	60.1 (10.6)	59.7 (6.5)	3.1 (1.5)	3.9 (0.9)	1.5T	3 mm	0 mm
**Seppi et al. 2006**	Putamen*	46	15	20	11	100.0	95.0	64.0 (5.5)	62.0 (8.3)	60.0 (5.8)	3.5 (2.1)	3.9 (1.8)	1.5T	3 mm	0 mm
**Seppi et al. 2006**	posterior putamen	46	15	20	11	100.0	100.0								
**MCP**															
**Chung et al. 2009**	MCP	32	10	12	10	91.7	100.0	63.6 (8.25)	65.7 (10.88)	62.1 (9.77)	2.0 (1.1)	2.5 (1.8)	1.5T	6 mm	1 mm
**Nicoletti et al. 2006**	MCP	63	16	16	15	100.0	100.0	64.7 (5.1)	61.0 (7.7)	67.5 (6.0)	4.9 (4.0)	7.5 (5.8)	1.5T	5 mm	1 mm
**Pons**															
**Ito et al. 2007**	pons	61	20	21	20	70.0	70.0	61.0 (9)	62.0 (11)	62.0 (11)	4.0 (2.0)	10.0 (8.0)	3T	2 mm	0.6 mm
**Cerebellum**															
**Ito et al. 2007**	cerebellum	61	20	21	20	60.0	87.5	61.0 (9)	62.0 (11)	62.0 (11)	4.0 (2.0)	10.0 (8.0)	3T	2 mm	0.6 mm
**Sako et al. 2016**	cerebellum	47	11	36	NA	91.0	64.0	60.0 (7.3)	61.0 (8.2)	NA	3.1 (2.3)	4.7 (4.5)	1.5T and 3T	6 mm	1.5 mm
**Nucleus Caudatus**															
**Nicoletti et al. 2006**	caudate nucleus	63	16	16	15	75.0	93.7	64.7 (5.1)	61.0 (7.7)	67.5 (6.0)	4.9 (4.0)	7.5 (5.8)	1.5T	5 mm	1 mm
**Globus Pallidus**															
**Nicoletti et al. 2006**	globus pallidus	63	16	16	15	62.5	93.7	64.7 (5.1)	61.0 (7.7)	67.5 (6.0)	4.9 (4.0)	7.5 (5.8)	1.5T	5 mm	1 mm

n = number, MSA-P = parkinsonian variant of Multiple system atrophy, PD = Parkinson´s disease, HC = healthy controls, NA = not applicable, MRI = magnetic resonance imaging, T = tesla,

* = these studies were included in Analysis

**Fig 2:**
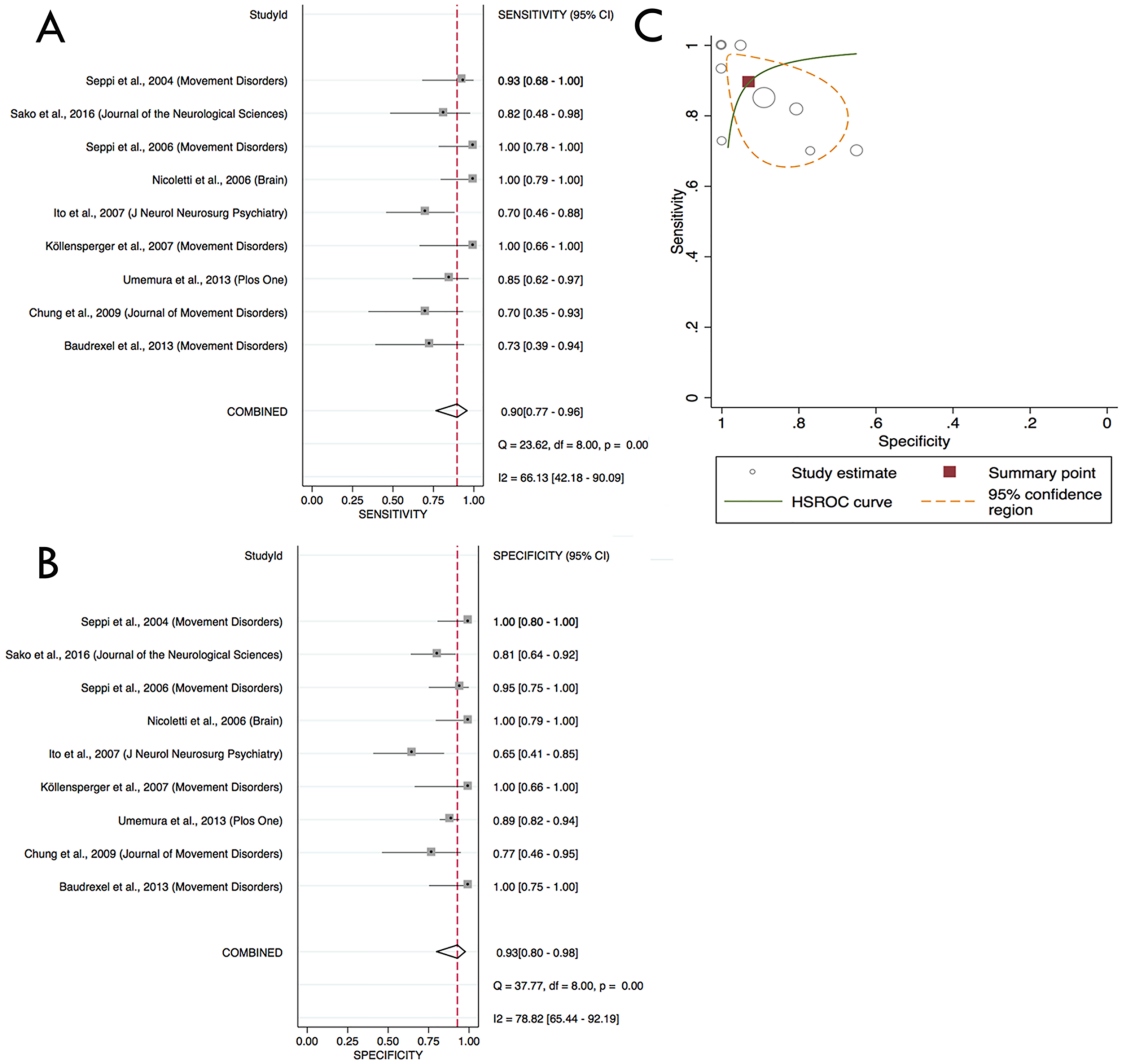
A: Overall sensitivity and B: overall specificity of putaminal diffusivity to discriminate MSA-P from PD. C: hierarchical summary receiver-operating characteristic (HSROC) curve plot demonstrating putaminal diffusivity accuracy to distinguish between MSA-P and PD in all 9 studies. The summary point shows overall sensitivity and specificity over the studies.

A sufficient number of studies to conduct a meta-analysis was published only for overall putaminal diffusivity measurements. Data from 127 MSA-P patients and 262 PD patients were analysed. Overall sensitivity was 90% (95% confidence interval: 76.7%–95.8%) and an overall specificity was 93% (95% confidence interval: 80.0%–97.7%) to discriminate MSA-P from PD patients (Fig 2). Excellent positive and negative likelihood ratios of 12.43 (3.97–38.92) and 0.11 (0.05–0.28), respectively, were observed. There was substantial between-study heterogeneity as suggested by I^2^ score of 66.13 and 78.82 for sensitivity and specificity, respectively.

Results of DWI measurements in five additional brain regions were reported in the literature. Nicoletti et al. were able to discriminate MSA-P from PD with a sensitivity and specificity of 100% based on measuring diffusivity in the MCP. [14] Following this approach, Chung et al. were able to replicate the excellent specificity but found a lower sensitivity to differentiate MSA-P from PD (sensitivity and specificity of 92% and 100%, respectively). [13] Analysis of the caudate nucleus also revealed a sensitivity of 75% and a specificity of 94% comparing MSA-P and PD. [14] Another study also measured diffusivity in the globus pallidus where sensitivity and specificity reached 63% and 93% for discriminating MSA-P from PD. [14] Ito et al. performed analyses in the pons to differentiate MSA-P from PD with a sensitivity and specificity of 70% each. [18] Two further studies described measurements in the cerebellum. Sensitivity ranged from 60% to 91% and specificity from 88% to 64%. [18,19] Moreover, magnetic field strength, slice thickness and interslice gap varied between studies. Two studies used 3T field strength, [15,18] other six studies used 1,5T field strength [13,14,16,17,20,21] and one used both.[19] Slice thickness varied from 2 mm to 6 mm and interslice gap varied from 0 mm up to 1.5 mm. A detailed overview is provided in Table 1.

All studies used established diagnostic criteria as a reference standard. Five out of nine studies included only probable MSA according to the current Consensus Criteria, the other studies included probable and possible MSA cases.[4] Five out of nine studies (56%) reported the method of patient recruitment and six out of nine (67%) reported the blinding status.

## Discussion

This meta-analysis shows that assessment of putaminal diffusivity on high-field DWI is a useful imaging technique to discriminate MSA-P from PD with overall sensitivity of 90% and overall specificity of 93%.

Putaminal diffusivity changes in MSA-P seem to correspond to prominent neuronal loss in the putamen in this disorder. Since diffusivity is based on hydrogen motility, structural damage in the putamen would lead to enhanced diffusivity [22] which can indeed be detected already in early disease stages in MSA-P patients. [16,20,23] Although normal aging may also affect diffusion tensor imaging, [24] none of the studies assessed here was confounded by age differences between study groups.

Our meta-analysis showed substantial between-study heterogeneity. Several factors might contribute to this variability: (1) slice thickness and interslice gap varied considerably between the studies included in this meta-analysis and it appears natural that a thinner slice thickness and a smaller inter-slice gap provides better diffusivity read-outs. (2) Differences in size and placement of the region of interests (ROIs) may have also influenced results. While some authors determined diffusivity in the putamen others restricted their ROIs to the posterior putamen. A standardized placement of the ROIs could be helpful in harmonizing results among different study sites. (3) Another potential source of variability arise from the used ROI placement procedure, i.e. automated atlas based definition of ROI or manual delineation of the ROI. In the present meta-analysis eight out of nine studies used manually placed ROIs and it remains to be studied which method provides better test-retest reliability. Magnetic field strength varied between the included studies from 1.5 T to 3 T. In total six studies used 1.5 T [13,14,16,17,20,21]], two used 3T [15,18]] and one used both field strengths [19]]. However, it is unlikely that this circumstance influenced the results of our meta-analysis since diffusion tensor do not depend directly on the magnetic field and can thus be measured and directly compared between high- and low-field acquisitions. In fact, water diffusion in a given space is the same at 1.5, 3.0 and even 7.0 T [25].

It is worth mentioning that none of the patients in any study had a post-mortem confirmed diagnosis. As clinical diagnostic certainty increases with disease progression, most of the studies have included patients in advanced disease stages, thus making the clinical diagnosis of patients more reliable. Other studies, having also included patients in earlier disease stages and followed patients clinically for at least 1 year to optimise diagnostic certainty. [20,21] Nevertheless, we cannot rule out clinical misclassification in some instances, but this is an inherent problem in clinical biomarker research in neurodegenerative parkinsonism. However all studies analysed here have used established diagnostic criteria for diagnosis of MSA-P. [4]

Three studies compared the diagnostic value of striatal ADCs or putaminal diffusivity to either dopamine D2 receptor binding IBZM-SPECT ([132-I]-iodobenzamide—single-photon emission computed tomography), [21] cardiac MIBG ([132-I]-meta-iodobenzylguanidine uptake) [17] or 18-Fluorodeoxyglucose positron emission tomography (FDG-PET). [15] Putaminal diffusivity measures were more accurate as compared with IBZM-SPECT, cardiac MIBG and FDG-PET imaging.

In summary, DWI is easy to implement in routine MRI protocols. Based on this meta-analysis, putaminal diffusivity on DWI has excellent sensitivity and specificity in distinguishing MSA-P from PD in clinically established cases, nevertheless, these results must be considered sober. Standardized MRI Protocols, harmonized DWI sequenzes and ROIs are needed to increase the inter-scanner and inter-site comparability. Further studies which directly compare different ROI placements are another important area of future research, also further studies comparing different methods are needed. Finally, all studies included in this meta-analysis analysed patients with an established clinical diagnosis, hence, multicenter imaging studies in patients with newly diagnosed parkinsonism with harmonized MR protocols and long-term clinical follow-up are highly warranted to inform us about the diagnostic accuracy of DWI in early disease stages when clinical diagnosis is often inaccurate.

## Supporting information

S1 File Search strategy. (DOCX)

S1 Checklist Prisma checklist. (DOC)

S1 Table Quadas. (XLSX)
