# Supplementary material for: Diffusion-weighted MRI distinguishes Parkinson disease from the parkinsonian variant of multiple system atrophy: A systematic review and meta-analysis
Source: PLoS One. 2017 Dec 29;12(12):e0189897. doi: 10.1371/journal.pone.0189897 (PMC5747439; doi:10.1371/journal.pone.0189897)
Supplement: S1 File — (DOCX) [file pone.0189897.s001.docx]

S1 File. Search Strategy for Pubmed, Clarivate Analytics® Web of Science® Core Collection.

*(“multiple system atrophy” OR MSA OR “olivopontocerebellar atrophy” OR OPCA OR “striatonigral degeneration” OR SND OR “Shy-Drager syndrome”) AND (“magnetic resonance imaging” OR MRI OR diffusion* OR diffusivity* OR DWI OR DTI OR ADC or “apparent diffusion coefficient”).*

1. Multiple system atrophy
2. MSA
3. Olivopontocerebellar atrophy
4. OPCA
5. Striatonigral degeneration
6. SND
7. Shy-Drager syndrome
8. 1 OR 2 OR 3 OR 4 OR 5 OR 6 OR 7
9. Magnetic resonance imaging
10. MRI
11. Diffusion*
12. Diffusivity*
13. DWI
14. DTI
15. ADC
16. apparent diffusion coefficient
17. 9 OR 10 OR 11 OR 12 OR 13 OR 14 OR 15 OR 16
18. 8 AND 18
